# Self-rated health, socioeconomic status and all-cause mortality in Chinese middle-aged and elderly adults

**DOI:** 10.1038/s41598-022-13502-9

**Published:** 2022-06-03

**Authors:** Yayun Fan, Dingliu He

**Affiliations:** Department of Clinical Nutrition, The Fourth Affiliated Hospital of Nantong University, The First People’s Hospital of Yancheng, No. 166, Yulong West Road, Yancheng, 224001 Jiangsu Province People’s Republic of China

**Keywords:** Health care, Risk factors

## Abstract

Our study aims to investigate the association between SRH and all-cause mortality, and to investigate whether the SRH-mortality association varies across different socioeconomic status (SES) groups among middle-aged and older Chinese adults. We used data from China Health and Retirement Longitudinal Study (CHARLS), including 11,762 participants for the final analysis. Cox proportional hazards regression was conducted to investigate the association between SRH status and subsequent mortality. There were 724 death events occurred. The results were shown that fair/poor SRH participants tend to die than better SRH peers (HR 1.46, 95% CI 1.12–1.91). The association only occurred in those with rural residency (HR 1.46, 95% CI 1.05–2.04), those who were literate (HR 1.65, 95% CI 1.17–2.33), those with above-average household income (HR 1.95, 95% CI 1.15–3.29) and those working in agriculture and below (HR 1.38, 95% CI 1.02–1.88). In conclusion, worse SRH may be a predictor of all-cause mortality among middle-aged and elderly Chinese, especially in people with rural residency, literacy, above-average household income and working in agriculture and below.

## Introduction

The world is experiencing the continuous aging due to an increasing life expectancy and low fertility rate^1^. It was estimated that the people aged 65 and older would account for one-sixth of the total world population by 2050, arising from one-eleventh in 2019^2^. In China, the number of senior citizens is also climbing so quickly because China’s “baby boomers” born in the 1950s and 1960s are stepping into old age^3^. In 2010, 111 million elderly aged over 65 lived in China and the number is predicted to rise to 400 million in 2050, which accounts for 26.9% of the national population^4^. Unfortunately, this substantial demographic transition contributes to the surge in the morbidity and mortality of middle-aged and elderly-related diseases, such as ischaemic heart disease, stroke, chronic obstructive pulmonary disease, lung and liver cancer^5^. Therefore, more mortality indicators should be discovered for people aged 45 and older. Although several hematology biomarkers have been reported to predict all-cause mortality^6,7^, they are difficult to be applied in health survey because of high costs in detection and difficulties in extracting blood samples. Identifying non-invasive and convenient measures to discover individuals at high-risk of mortality in the general population is warranted.

Self-rated health (SRH), a personal perception of individuals’ health status, has been widely recommended to be used in the health survey by World Health Organization (WHO)^8^. Although it is easily measured, SRH has been suggested as a strong predictor of vascular events^9,10^, mental disorders^11^ and functional ability^12^. In recent years, the association between SRH and all-cause mortality has been reported in different cohorts^13,14^, but inconsistent results were also shown, which could be reflected by the presence of such significant associations in populations with different demographic characteristics^15–18^. Thus, this association should be further verified among the Chinese population.

In addition, as a subjective evaluation of health, SRH can be influenced by the socioeconomic status (SES) factors, which were consisted of urban/rural residency, income level and education status^19,20^. Indeed, SES-related differences have been shown to modify the association between SRH and all-cause mortality^21,22^. However, most studies mentioned above were conducted in developed countries where the study populations had relatively high socioeconomic classes. Moreover, conflicting results were also observed in the sub-groups of different SES status. For example, the education-related difference in SRH may further modify the association between SRH and death events. Specifically, previous works of literature have shown that a stronger association between SRH and all-cause mortality exists in higher rather than lower educated individuals^23,24^. By contrast, another two studies argued that the aforementioned relationship for education was similar^25,26^. Unfortunately, few studies have evaluated the predictive value of SRH for overall mortality in the low-to-middle income countries^16^, and the role that SES plays in the association between SRH and all-cause mortality is still unclear.

To fill this gap, we used data from China Health and Retirement Longitudinal Study (CHARLS) to investigate the association between SRH and all-cause mortality in middle-aged and older Chinese, and to investigate whether the SRH-mortality association varies across different SES groups.

## Materials and methods

### Study population

In the current study, we used 4-year follow-up data from CHARLS, a population-based longitudinal cohort study of middle-aged and elderly adults conducted in China. Details of the CHARLS study design and respondents were described elsewhere^27^. In the baseline survey (wave 1), conducted between June 2011 and March 2012, 17,708 participants from 10,257 households were recruited. After the baseline interview, subsequent three follow-ups were conducted in 2013–2014 (wave 2), 2014–2015 (wave 3) and 2015–2016 (wave 4), respectively. CHARLS was approved by the Institutional Review Board of Peking University and all methods were performed in accordance with the relevant guidelines and regulations. All respondents provided written informed consent. If the respondent was illiterate, he/she would press the fingerprint after the interviewer dictated the content of the informed consent.

**Figure 1:**
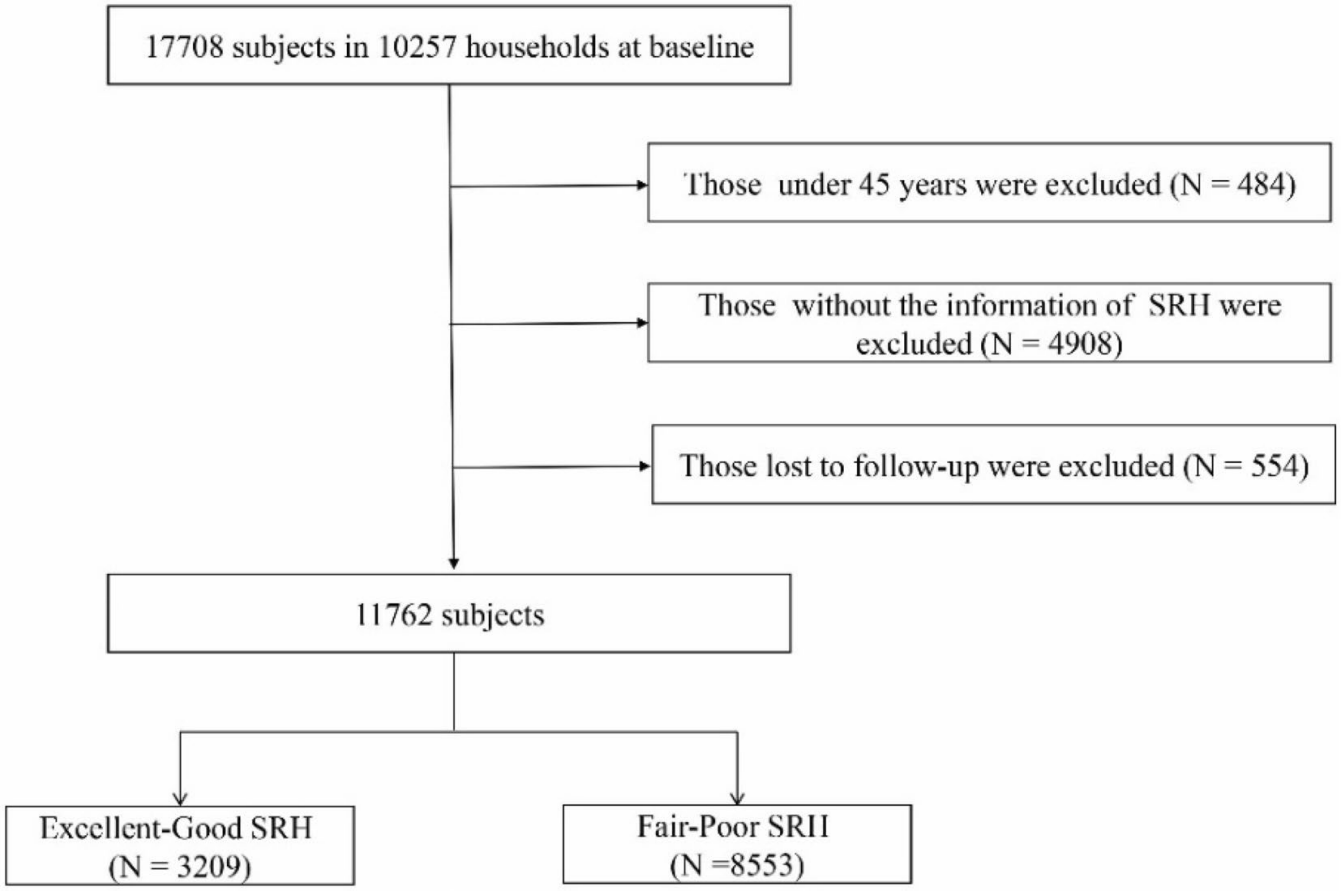
Flowchart of subject selection.

According to the analytical purposes, we excluded participants with the following criteria: (1) individuals under 45 years (*n* = 484), (2) individuals without information of SRH at baseline (*n* = 4908), (3) individuals who were lost to follow-up (*n* = 554). Finally, 11,762 subjects were included in the analysis (Fig. 1).

### Assessment of self-rated health

Self-rated health status was acquired by the question: “Currently, how would you evaluate your current health condition?”, with response options ranging from “Excellent”, “Very good”, “Good”, “Fair” to “Poor”. In our study, we defined SRH by dichotomizing answers into “excellent to good” versus “fair to poor” for subsequent analysis^28^.

### Assessment of mortality

Mortality was determined by the interview status (alive or dead) of participants in waves 2, 3 and 4. The information of the interview date could be obtained from all three follow-ups, but the exact death time was only available in wave 2. If participants had survived during the observation period, the survival time was calculated as the interval between two surveys. If death events occurred, the survival time was the interval from the date of wave 1 to the date of participants’ death or the median time from the date of the first interview to the wave with death record.

### Covariates

We used data from the baseline survey to define covariates. In the current study, age, sex, SES, smoking status, drinking status, body mass index (BMI) and self-reported history of chronic diseases were included. SES was indicated by living place, education status, income level and occupation status. The living place was categorized as urban and rural. Education status was dichotomized as illiterate versus literate. Income level was classified into above-median household income or below-median household income. Occupation status was categorized as agriculture work and below (including agriculture work, unemployed, never work and retired without pension) and non-agriculture work (including non-agricultural work for wages, non-agricultural self-employed work, non-agricultural work without pay for a family business and retired with pension). Smoking status was dichotomized as ever versus never, and drinking status as ever *versus* never. BMI was calculated through weight (kg) divided by height squared (m^2^). Chronic diseases were all dichotomized as yes versus no, which included hypertension, high blood sugar/diabetes, cancer, chronic lung disease, stroke, heart problems, arthritis, dyslipidemia, liver disease, kidney disease, digestive disease, asthma, memory-related disease and emotional, nervous or psychiatric disorders.

### Statistical analysis

Continuous variables were expressed as mean ± standard deviation (SD) or median (25th-75th percentile) and categorical variables were presented as count (percentage). The differences in baseline characteristics across SRH levels were compared by t test or Kruskal–Wallis test for continuous variables and by the Pearson chi-square test for categorical variables. Kaplan–Meier curve was used to show the incident rates of mortality by SRH levels. The association between SRH and all-cause mortality was evaluated by hazard ratios (HRs) and 95% confidence intervals (CIs) using Cox proportional hazards regression models. In the Cox model, the endpoint event was death and the censored event was designated to survive. And the specific calculation of survival time could be found in the section of the assessment of mortality. We also evaluated the moderating effects of SES factors on this association through repeated stratified analyses by living place, education status and income level. Furthermore, considering the death cause profile between middle-aged and elderly adults is different, as well the gender difference is also very crucial for SES and mortality because of the 'men-women health paradox', so we additionally conducted the subgroup analyses on the association of baseline SRH with mortality for age and gender. All statistical analyses were conducted with SAS 9.4 (SAS Institute Inc., Cary, NC, USA) and a two-sided *P* value < 0.05 was deemed as statistical significance.

## Results

Of 11,762 participants, 3209 (27.28%) individuals rated their health status as excellent, very good or good, and 8553 (72.72%) subjects responded SRH as fair or poor. Participants with worse SRH status were more likely to be women. Worse SRH was more prevalent in the elderly, rural residents, participants working in agriculture and below, non-smokers and non-drinkers. In addition, individuals with fair or poor SRH status had higher prevalence rates of all chronic diseases (Table 1).

**Table 1:** Characteristics of the study population.

Variables (%)	All participants	Self-rated health		*P* value
		Excellent to good	Fair to poor	
*N* (%)	11,762	3209 (27.28)	8553 (72.72)	
Age (years)	59.22 ± 9.82	57.83 ± 9.66	59.73 ± 9.82	< 0.0001
**Sex, *N* (%)**				
Male	6704 (57.00)	2056 (64.07)	4648 (54.34)	< 0.0001
Female	5058 (43.00)	1153 (35.93)	3905 (45.66)	
**Living place, *N* (%)**				
Urban	4602 (39.13)	1421 (44.28)	3181 (37.19)	< 0.0001
Rural	7160 (60.87)	1788 (55.72)	5372 (62.81)	
**Education, *N* (%)**				
Illiterate	2983 (25.36)	660 (20.57)	2323 (27.16)	< 0.0001
Literate	8779 (74.64)	2549 (79.43)	6230 (72.84)	
**Income, *N* (%)**				
Below-median	4510 (47.61)	993 (38.55)	3517 (51.00)	< 0.0001
Above-median	4962 (52.39)	1583 (61.45)	3379 (49.00)	
**Occupation, *N* (%)**				
Agricultural work and below	7955 (68.20)	1850 (58.23)	6105 (71.93)	< 0.0001
Non-agricultural work	3709 (31.80)	1327 (41.77)	2382 (28.07)	
BMI, kg/m^2^	23.0 (20.7–25.6)	23.2 (21.2–25.6)	22.8 (20.6–25.5)	< 0.0001
Smoking, *N* (%)	5541 (47.16)	1605 (50.03)	3936 (46.08)	0.0001
Drinking, *N* (%)	6440 (54.87)	1947 (60.71)	4493 (52.68)	< 0.0001
Diabetes, *N* (%)	731 (6.27)	104 (3.26)	627 (7.41)	< 0.0001
Cancer, *N* (%)	130 (1.11)	24 (0.75)	106 (1.25)	0.0223
Lung disease, *N* (%)	1307 (11.16)	195 (6.08)	1112 (13.08)	< 0.0001
Heart disease, *N* (%)	1455 (12.45)	174 (5.43)	1281 (15.10)	< 0.0001
MRD, *N* (%)	270 (2.31)	27 (0.84)	243 (2.85)	< 0.0001
Arthritis, *N* (%)	4026 (34.33)	650 (20.29)	3376 (39.61)	< 0.0001
Dyslipidemia, *N* (%)	1135 (9.85)	181 (5.72)	954 (11.42)	< 0.0001
Liver disease, *N* (%)	460 (3.94)	56 (1.75)	404 (4.77)	< 0.0001
Kidney disease, *N* (%)	704 (6.03)	80 (2.50)	624 (7.37)	< 0.0001
Digestive disease, *N* (%)	2595 (22.15)	391 (12.20)	2204 (25.89)	< 0.0001
Asthma, *N* (%)	584 (4.99)	64 (2.00)	520 (6.11)	< 0.0001
Hypertension, *N* (%)	3161 (27.05)	577 (18.04)	2584 (30.45)	< 0.0001
ENP, *N* (%)	181 (1.55)	17 (0.53)	164 (1.93)	< 0.0001
Stroke, *N* (%)	336 (2.87)	33 (1.03)	303 (3.56)	< 0.0001

MRD, memory-related disease; ENP, emotional, nervous, or psychiatric problems. Values were presented as mean ± SD, n (%) or median (25th–75th percentile).

**Figure 2:**
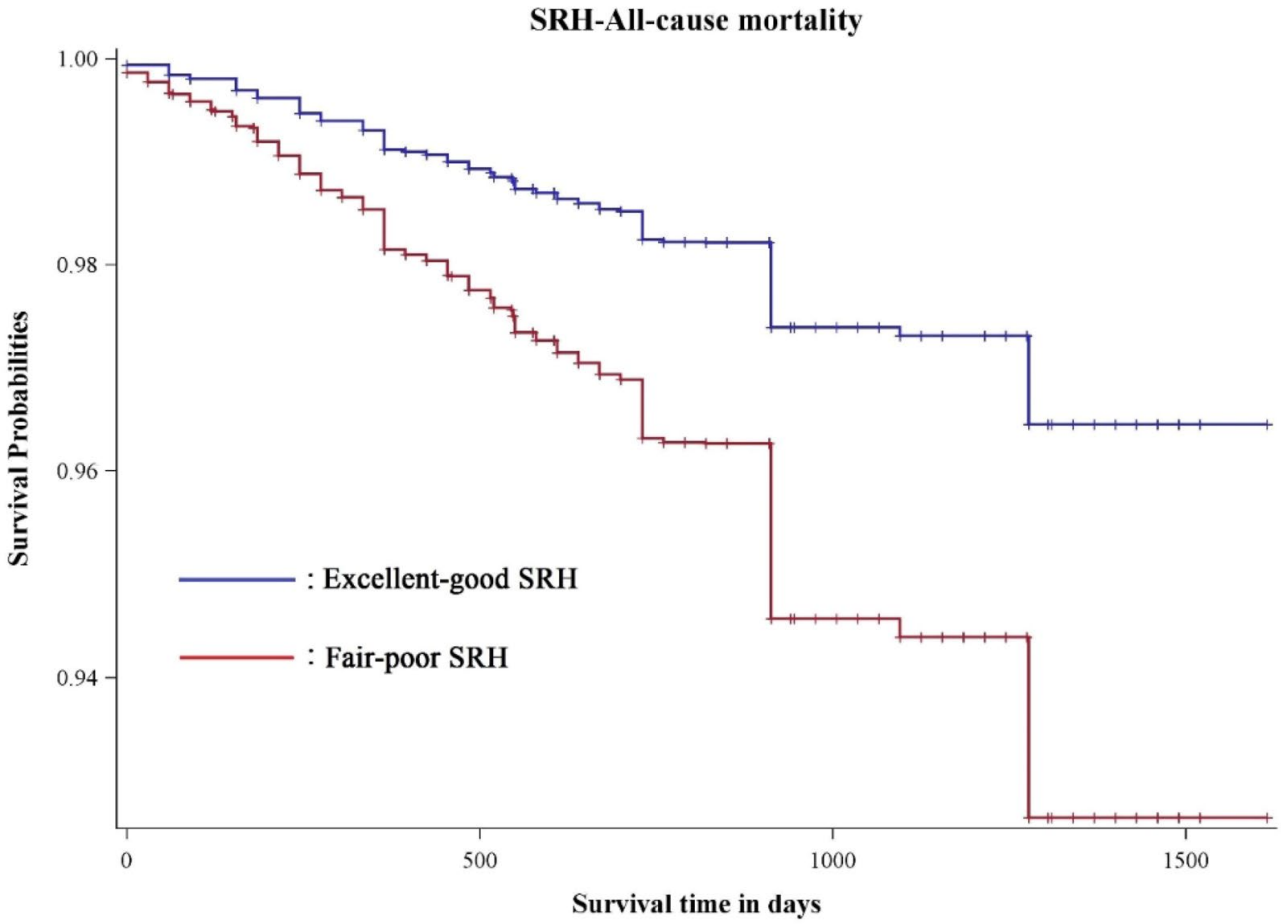
Kaplan–Meier curves for the cumulative risk of mortality by SRH levels.

During the 4-year follow-up, 724 death events were reported, with a mortality rate of 6.16%. Subjects with fair-poor SRH status possessed a higher mortality rate than those with excellent-good SRH status (Fig. 2). Relative to individuals with better SRH status, fair-poor SRH status was associated with an increased risk of total mortality in the unadjusted model (HR 2.11, 95% CI 1.73–2.59). After fully adjusted for a series of covariates (Model 5), the HR (95% CI) was attenuated to 1.44 (1.10–1.88) (Table 2).

**Table 2:** Association of SRH with all-cause mortality risk.

All-cause mortality	SRH scale	
	Excellent—Good	Fair—Poor
Events, n (%)	110 (3.43)	614 (7.18)
Unadjusted	1.00 (ref)	2.11 (1.73, 2.59)
Model 1 ^a^	1.00 (ref)	1.47 (1.16,1.87)
Model 2 ^b^	1.00 (ref)	1.47 (1.16,1.87)
Model 3 ^c^	1.00 (ref)	1.47 (1.16,1.87)
Model 4 ^d^	1.00 (ref)	1.45 (1.11, 1.89)
Model 5 ^e^	1.00 (ref)	1.44 (1.10, 1.88)

Values were presented as hazard ratios (95% confidence interval). SRH, self-rated health

^a^Model 1: adjusted for age, sex, smoking status, drinking status, BMI, hypertension, Emotional, nervous, or psychiatric problems (ENP), dyslipidemia, diabetes, heart problems, cancer, chronic lung disease, memory-related disease, kidney disease, liver disease, arthritis, digestive disease and asthma, ^b^Model 2: Model 1 + living place, ^c^Model 3: Model 2 + education status, ^d^Model 4: Model 3 + income level, ^e^Model 5: Model 4 + Occupation.

**Figure 3:**
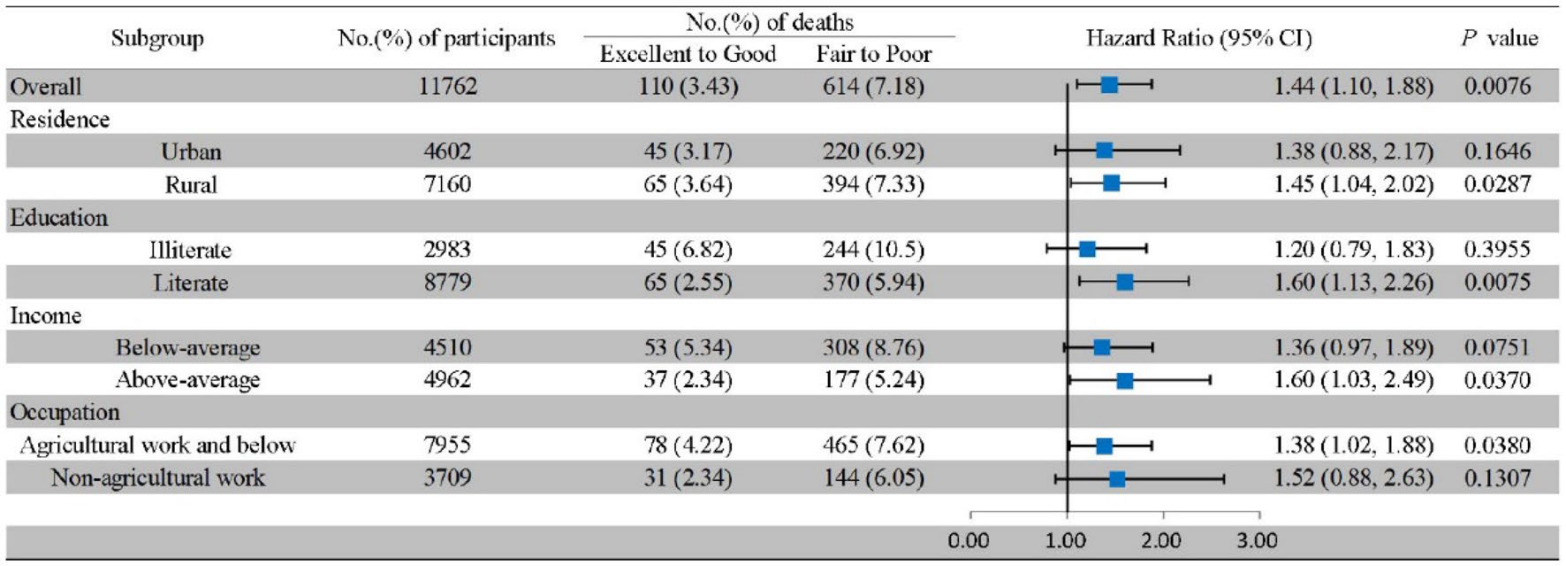
Stratified analyses of SES factors in the association of SRH with all-cause mortality. *Notes* Model was adjusted for age, sex, smoking status, drinking status, BMI, hypertension, Emotional, nervous, or psychiatric problems (ENP), dyslipidemia, diabetes, heart problems, cancer, chronic lung disease, memory-related disease, kidney disease, liver disease, arthritis, digestive disease and asthma, living place, education status and income level.

In stratified analyses by SES factors, the positive association between fair-poor SRH and all-cause mortality risk was only detected in those with rural residency (HR 1.45, 95% CI 1.04–2.02), those who were literate (HR 1.60, 95% CI 1.13–2.26), those with above-average household income (HR 1.60, 95% CI 1.03–2.49) and those working in agriculture and below (HR 1.38, 95% CI 1.02–1.88; Fig. 3). Besides, we additionally performed stratified analyses by age and gender, and the results showed that the impact of fair-poor SRH on all-cause mortality only occurred in those who were under 60 years old (HR 1.97, 95% CI 1.07–3.64) and men (HR 1.52, 95% CI 1.10–2.09; Supplemental Figure S1).

## Discussion

Based on 11,762 middle-aged and elderly adults in China, our study found that fair-poor SRH status was associated with an elevated risk of all-cause mortality, especially among people with literacy, rural residency and above-average household income.

The relationship between SRH and overall mortality had been evaluated in previous studies^13,14^. However, the contradictory results were also reported in a limited number of studies^17,18^. McCallum et al. found that poor SRH status was not associated with mortality among 1050 elderly people, particularly in the fully adjusted model^17^. Likewise, a prospective cohort of 645 people aged 60 and over also indicated that SRH was not a risk marker of mortality in the multivariate survival model^18^. Nevertheless, it is worth noting that the sample sizes of these two studies were relatively small and were all conducted in Western countries. In the West, the leading cause of death is cardiovascular disease, but in China, it is cancer^29^. Besides, the dimensions in scales they used to evaluate SRH were also different from ours.

Overall, our nationwide and large-scale study proved that this association still existed among middle-aged and elderly Chinese adults. In stratified analyses by SES factors, our study demonstrated the positive associations between fair-poor SRH and overall mortality only in participants with rural residency, literacy and above-average household income. Actually, high education attainment and income as the conventional indicators of well SES, have also been found to strengthen the association among the US population^23^. One possible explanation is that people with higher levels of education and income are more likely to have health-related knowledge and access to health services, which helps them to perceive their health status more accurately^23^. On the contrary, older residents with low SES status are more superstitious and have poor health literacy so that they always misinterpret their body feelings^30,31^. For example, those people always reported their health state as fair even in the cases of serious diseases because they argued that a negative evaluation would bring misfortune and bad health^32,33^. Furthermore, it should be noted that the huge gaps in economic development level, environmental quality and medical resources between urban and rural areas still exist in China^34^. Thus, we additionally used urban–rural residency as an indicator of SES in the current study, and we demonstrated that worse SRH was associated with an elevated risk of death from all-cause only in rural residents. It has been reported that there is an uneven distribution of the health workforce in China^35^. Worse quality and quantity of health workers in the country prevents the rural residents from receiving timely and effective cures when suffering from accidental injury. In addition, compared with urban areas, the prevalence, awareness, treatment and control of chronic diseases, such as stroke, diabetes and cardiovascular disease, in rural places are obviously worse, which extremely aggravates the mortality risk of rural residents^36^.

The potential mechanisms linking SRH and all-cause mortality could be attributed to several reasons, which include the characteristic of SRH, genetic factors, psychological distress and unhealthy behaviors^37^. Firstly, SRH is a personal subjective assessment based on their objective health status, and poor health could affect both the reporting of SRH and the risk of death. Secondly, a recent genome-wide association study (GWAS) found that some genes were associated with both SRH and longevity. Specifically, the gene scores for several diseases which increase the new-onset risk of death (e.g., ischemic heart disease, macrovascular stroke and type 2 diabetes) were also associated with SRH^38^. Thirdly, worse SRH, as a sensitive indicator of health, could synthetically reflect their poor physical status and social support, which may limit the contact with health services and induce mental stress^39^. Stress is known to be associated with low-grade chronic inflammation and elevated level of pro-inflammatory cytokines^40^. While deregulation of inflammation would further contribute to the occurrence of cardiovascular disease^41^ and cancer^42^, which in turn increase the risk of mortality. In UK Biobank, scientists also found that neuroticism and stress (e.g., depression and anxiety) were weakly/moderately associated with all-cause mortality^43^. As well, the fair or poor SRH status has been considered as the consequence of physical inactivity^44^. Moreover, physical inactivity is well known as the risk factor of obesity and chronic non-communicable diseases (CNCDs)^45^, all of which were recognized as the leading causes of mortality.

Limitations of the present study should be considered. Firstly, the duration of our study is relatively short so that a small number of death events were observed, which may underestimate the association between worse SRH and all-cause mortality. The association should be further validated with a long-term follow-up. Secondly, the data of SRH were only recorded at baseline, while SRH would change with age. Future studies should investigate the association between SRH change and the risk of mortality. Thirdly, although we have adjusted for many potential covariates, several confounding residuals, such as the effect derived from endogeneity between lifestyle (smoking or drinking) and SRH, cannot be completely rolled out, which may prevent us from discovering stronger associations. Lastly, the information on specific-cause mortality was unavailable in CHARLS so that we were not able to investigate the predictive value of SRH on specific-cause of death. Such variables should be added to subsequent follow-ups, which may help researchers to expand current results among the Chinese population.

## Conclusions

In summary, our study provides the evidence that SRH could serve as a predictor of all-cause mortality among the middle-aged and elderly Chinese population. Considering the practical application, a cost-effective and non-invasive tool, such as SRH, could be used to monitor high-risk individuals of mortality, particularly in people with rural residency, literacy and above-average household income. Meanwhile, more targeted and intensive health care should be taken to decrease the probability of death in the high-risk population. Furthermore, our finding emphasizes the importance of the cultivation of rural medical staff and recollection of medical resources to reduce the urban–rural disparity, which may change the health outcomes faced by rural residents and effectively help to achieve the government aim of a Healthy China by 2030.

## Supplementary Information

Supplementary Figure 1.

## Data Availability

The datasets analysed during the current study are available in the CHARLS repository, www.g2aging.org.
