# Supplementary material for: Self-rated health, socioeconomic status and all-cause mortality in Chinese middle-aged and elderly adults
Source: Sci Rep. 2022 Jun 3;12:9309. doi: 10.1038/s41598-022-13502-9 (PMC9166789; doi:10.1038/s41598-022-13502-9)
Supplement: Supplementary file 1 — Supplementary Figure 1. [file 41598_2022_13502_MOESM1_ESM.docx]

**Supplementary materials**

**Self-rated health, socioeconomic status and all-cause mortality in Chinese middle-aged and elderly adults**

Yayun Fan and Dingliu He

Department of Clinical Nutrition, The Fourth Affiliated Hospital of Nantong University, The First People's Hospital of Yancheng, Yancheng, 224001, P. R. China

**
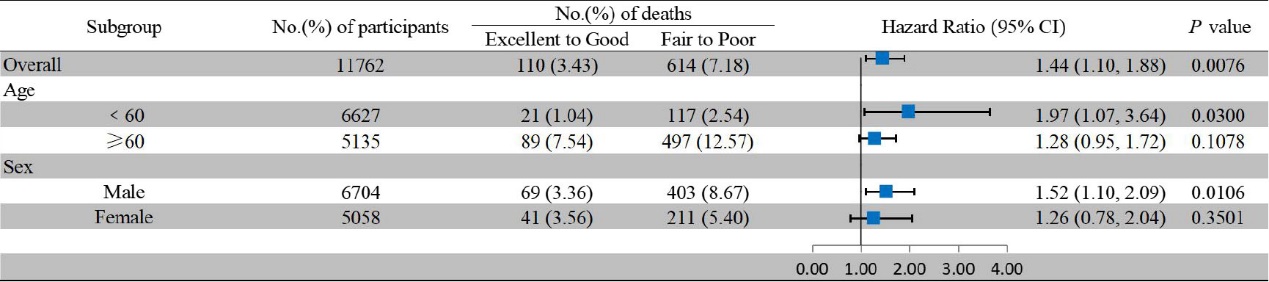
**

**Supplementary Figure 1** Stratified analyses of age and sex in the association of SRH with all-cause mortality
